# Metastatic Clear Cell Renal Cell Carcinoma Presenting as Acute Appendicitis

**DOI:** 10.7759/cureus.44155

**Published:** 2023-08-26

**Authors:** Oxana Ushakova, Keyvan Ravakhah

**Affiliations:** 1 Internal Medicine, MetroHealth Medical Center, Cleveland, USA; 2 Internal Medicine, Saint Vincent Charity Medical Center, Cleveland, USA

**Keywords:** rcc surveillance, recurrent rcc, metastatic rcc, renal cell carcinoma (rcc), atypical appendicitis

## Abstract

Renal cell carcinoma (RCC) is a malignant tumor arising from the epithelial cells of kidney tubules. It may metastasize to many sites; however, metastasis of RCC to the appendix is very rare. Renal cell carcinomas usually metastasize to the lungs, lymph nodes, bones, or liver. Metastasis usually occurs within three years after radical nephrectomy; however, there is evidence of RCC metastasis many years following nephrectomy.

## Introduction

Renal cell carcinoma (RCC) is a commonly known malignancy originating from kidney tubule epithelial cells, predominantly metastasizing to sites such as the lungs, lymph nodes, bones, and liver [1-3]. However, metastasis to the appendix is an extraordinary occurrence. This report presents the second documented case [4] of RCC metastasizing to the appendix, discovered in a 66-year-old man initially diagnosed with acute appendicitis. Remarkably, the metastasis was identified more than five years following a nephrectomy for RCC, highlighting the need for awareness of this rare metastatic site and potential long-term recurrence. RCC metastasis to other sites following many years (decades) after being successfully treated with surgery was documented by some other authors [5-8]. These cases, including ours, underscore that RCC patients cannot be considered fully cured even after successful resection and suggest a possible reconsideration of surveillance periods and practices. 

## Case presentation

The patient is a 66-year-old man with a past medical history of left-sided RCC pT2a followed by nephrectomy in 2017, chronic kidney disease stage 3b, benign prostate hyperplasia, and morbid obesity. He was a former smoker who quit 40 years ago. He denied alcohol or illicit drug use. The patient was evaluated in the emergency room complaining of a two-day history of sharp right lower quadrant pain (10/10), radiating to the right upper quadrant and being associated with nausea and decreased appetite. The patient denied having unintentional weight loss, fever, chills, dizziness, leg pain or swelling, or any other symptoms.

On the physical exam, there was no fever. He was hemodynamically stable and not tachycardic. His abdomen was tender in the right lower quadrant with no palpable masses. The obturator sign was positive. The rest of the physical exam was unremarkable.

**Figure 1:**
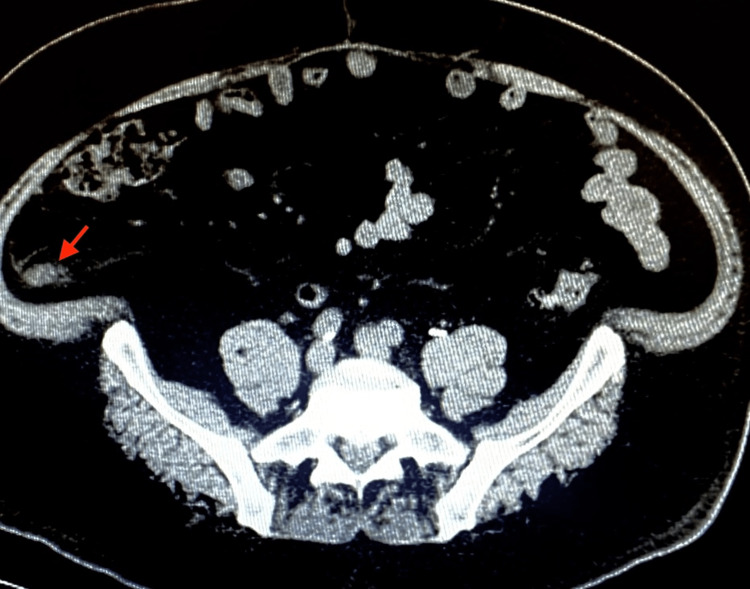
CT of the abdomen. Red arrow: the tip of the appendix reveals minimal inflammation.

CT scan of the abdomen demonstrated new fat stranding adjacent to the end of the appendix, suspicious for tip appendicitis, with no abscess (Figure 1). A complete blood count was normal.

The patient underwent an appendectomy with no intraoperative complications. Due to the patient’s obesity and potential anatomical difficulty, an open appendectomy was chosen. He was started on a course of metronidazole and ciprofloxacin.

**Figure 2:**
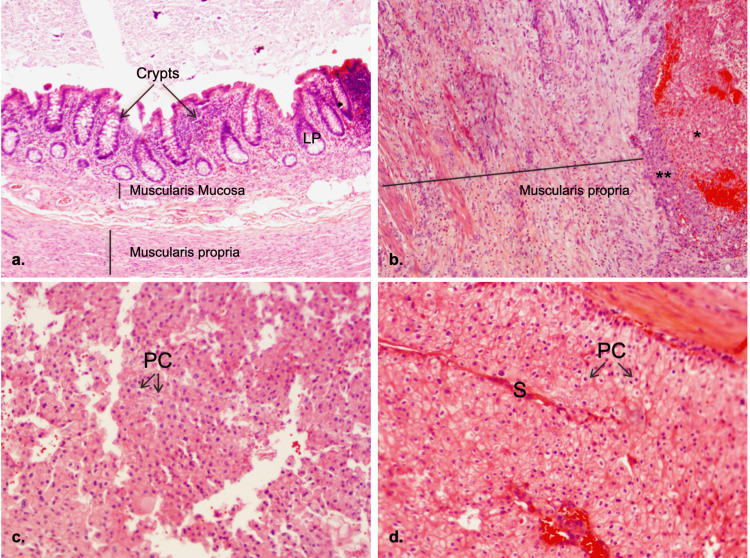
Tissue biopsy a. Appendix, normal appearing; a straight vertical line indicates the width of the layer. Hematoxylin and eosin stain, x40 magnification. b. Appendix tip metastatic tumor in the right muscle wall; *metastasis of clear cell renal cell carcinoma; **large lymphocytes with irregular nuclear contours and large nucleoli. Hematoxylin and eosin stain, x40 magnification c. Appendix metastases, tumor tip. PC: Polygonal cells with clear cytoplasm and centrally located nuclei. Hematoxylin and eosin stain, x100 magnification d. Renal cell carcinoma, specimen from the same patient five years ago. S: Delicate septa containing thin-walled blood vessels. PC: Polygonal cells with clear cytoplasm and centrally located nuclei. Hematoxylin and eosin stain, x100 magnification.

Two days following the surgery, the patient was discharged from the hospital in fair condition. The resected appendix was sent to pathology, where it turned out to be metastatic clear renal cell carcinoma (Figure 2). The patient was informed and underwent a PET scan with no evidence of metastatic lesions. He was recommended to follow up with an oncologist and get monitored annually with a PET scan. No further treatment was recommended at that time.

## Discussion

We report a rare case of RCC spreading to the appendix. This is the second such case in the medical literature. In the other case, a 65-year-old woman had sarcomatoid RCC metastasize to the small intestine, which resulted in a perforated appendix [4]. In Table 1, we compare these two cases against each other. RCC metastasis may spread hematogenously via the lymphatic system, direct extra-renal outgrowth, or peritoneal dissemination.

**Table 1:** The comparison of the presented case and the case reported by Liao X et al. in 2019

	Presented patient	Liao X et al. (2019)
Age	66	65
Cancer type	Clear-cell renal cell carcinoma	Clear-cell renal cell carcinoma with sarcomatoid features
Metastasis time after the primary tumor was found and being treated	6 years	1.5 years
Other metastasis	No	Lung, liver, bone
Required treatment	Only appendectomy	Appendectomy following radiotherapy
Outcome	The patient was instructed to follow up with an oncologist for an annual PET scan	Died one month after initiating treatment from the rapid progression of metastatic disease

Surgery is supposed to be a definitive treatment for non-metastatic disease; however, depending on the tumor grade and feature, adjuvant treatment might be indicated. There are no guidelines on early versus late metastatic disease treatment. In one study, the authors looked at the outcomes of the patients with late-relapse RCC treated with targeted therapies and concluded that these patients had prolonged survival that was favorable when compared to historical control. [9] Our patient underwent a PET scan looking for more metastatic lesions. Fortunately, the study was negative, but it created the dilemma of whether or not he should receive any kind of adjuvant therapy. This case is important not only from the location of metastatic disease but also from the time interval between the initial diagnosis and the subsequent recurrence of the disease. The initial, supposedly curative nephrectomy was performed more than five years before the current event. The frequency of surveillance is based on the stage, and the recommended surveillance period is usually no more than five years; however, the recurrence happens out of this window as well, and the further follow-up depends on the clinical indications [10].

## Conclusions

Renal cell carcinoma can metastasize to the appendix. In a patient with a remote history of RCC and clinical as well as radiological manifestations of acute appendicitis, metastasis from RCC should be kept in mind, and this should be communicated with the pathologist for proper staining and an accurate diagnosis. Given our case and the other reported cases with metastasis after decades of curative treatment mentioned above, patients with RCC still might develop distant metastasis and cannot be considered cured even five years from the initial diagnosis and successful resection of the confined tumor. Surveillance after the first recurrence remains unclear.
